# Longitudinal anemia status and risk for adverse outcomes in former smokers with COPD

**DOI:** 10.1186/s12931-024-03071-y

**Published:** 2024-12-19

**Authors:** Yukiko Kunitomo, Han Woo, Aparna Balasubramanian, Ashraf Fawzy, Cheng Ting Lin, Sarath Raju, Daniel C Belz, Meredith C McCormack, Kirsten Koehler, Nadia N Hansel, Nirupama Putcha

**Affiliations:** 1 Division of Pulmonary and Critical Care Medicine, Johns Hopkins University School of Medicine, 5501 Hopkins Bayview Circle, Baltimore, MD 21224 USA; 2 Department of Radiology, Johns Hopkins University School of Medicine, Baltimore, MD USA; 3 Environmental Health and Engineering, Johns Hopkins Bloomberg School of Public Health, Baltimore, MD USA

**Keywords:** COPD, Anemia, Comorbidity, Longitudinal analysis

## Abstract

**Background:**

Anemia is a prevalent comorbidity in COPD associated with increased morbidity. However, the significance of longitudinal anemia status and variation in anemia status trends over time in COPD are not known. Furthermore, individuals with COPD and smoking history often have multiple comorbidities, in particular cardiovascular disease. The objective of this study was to evaluate the association between longitudinal anemia status and COPD outcomes, accounting for comorbid cardiovascular disease.

**Methods:**

Serial hemoglobin measures and clinical outcomes were obtained in former smokers with moderate to severe COPD from two clinical studies over a 6-to-9-month period. In the first analysis, the association between repeated measures of time-varying anemia status and outcomes was assessed by generalized estimating equations adjusted for covariates including cardiovascular disease. In the second analysis, each participant’s anemia risk profile during the study period was characterized as high versus low anemia risk-growth rate. Mean differences in the progression of COPD outcomes over time between the two groups were assessed using a generalized linear mixed model. Effect modification by baseline coronary artery calcium (CAC) burden was explored.

**Results:**

There were 159 individuals with mean age of 66.5 years (± 8.3) and mean FEV_1_% predicted of 51.4% (± 17.0), of which 41% were ever-anemic during the study period. Repeated measures of anemia status were associated with higher St. George’s Respiratory Questionnaire (SGRQ) scores (β 2.5, 95% CI: 0.1,4.8, *p* = 0.04), lower 6-minute walk distance (6MWD) (β -38.6, 95% CI: -67.7,-7.4, *p* = 0.02), and higher rate of moderate-to-severe exacerbations over the prospective follow-up period (IRR 1.8, 95% CI: 1.1,2.8, *p* = 0.02). There was effect modification by CAC burden such that with higher burden the mean difference in COPD outcome by anemia status was greater for a subset of symptom scores. Participants with profiles of increasing anemia risk had higher estimated rates of decline in the FEV_1_% predicted and 6MWD and increase in SGRQ scores compared to those with stable or decreasing anemia risk.

**Conclusions:**

Longitudinal anemia status trends may be predictive of COPD disease trajectory. Anemia status by repeated measures analysis is associated with COPD morbidity with potentially stronger associations in the setting of high CAC burden.

**Supplementary Information:**

The online version contains supplementary material available at 10.1186/s12931-024-03071-y.

## Introduction

Individuals with COPD often have a high comorbidity burden associated with increased morbidity and mortality [1, 2]. Anemia is one such comorbidity with a prevalence between 7 and 33% among patients with COPD [3] and has been shown to be associated with increased severity of COPD exacerbations, higher rates of hospital re-admission, greater symptom burden, lower exercise capacity, and higher mortality [4–9]. These associations have primarily relied upon a single measurement of hemoglobin to define anemia. However, hemoglobin levels, and thus anemia status, are not static. Currently, how anemia status changes over time and the variability in anemia status trajectories in the COPD population are not known. Studies in the heart failure population have shown variable trends in hemoglobin or anemia status over a period of months to years after hospitalization for myocardial infarction or heart failure exacerbation, with longitudinal trends providing greater prognostic significance for health status, hospital readmission, and mortality compared to single baseline hemoglobin values [10, 11]. Therefore, understanding the association between longitudinal anemia status trends and clinical outcomes in the COPD population may provide greater insight into whether individuals who develop anemia or have fluctuating anemia status constitute a group at higher risk for adverse outcomes, and may better inform the approach to anemia management in COPD.

Furthermore, the association between anemia and COPD outcomes must be considered in the context of multimorbidity. Cardiovascular disease (CVD) is one of the most common comorbidities among individuals with COPD and a history of smoking [1]; there is a 2.5-fold increased risk of CVD [12] in COPD and up to one-third of patients with COPD die from CVD [13]. Epidemiological studies of anemia in the COPD population have demonstrated a high prevalence of CVD among anemic individuals compared to non-anemic individuals with COPD [5, 14, 15]. Specifically, coronary artery disease (CAD) in COPD is associated with increased symptoms (dyspnea) and reduced exercise capacity and CAD burden can be measured objectively by coronary artery calcium (CAC) burden on chest computed tomography [16, 17]. Accordingly, understanding the complex interplay between comorbid anemia and CVD is critical to fully capture the implications of each condition.

This study aimed to evaluate the association between longitudinal anemia status and COPD morbidity through assessment over time of (1) repeated measures of anemia status, and (2) anemia risk trajectories while also accounting for CVD comorbidity burden. We hypothesized that anemia will be associated with worse COPD morbidity by repeated measures analysis, while *development* of anemia, represented by increasing anemia risk, will be associated with worse COPD disease trajectory. In addition, we hypothesized that CAD severity measured by CAC burden [12, 13] to be an effect modifier of the association between longitudinal anemia status and COPD morbidity, with a more pronounced effect of anemia on outcomes in the setting of higher CAD burden. To this end, two clinical cohorts of individuals with history of smoking tobacco diagnosed with COPD in Baltimore, Maryland, the Clinical Trial of Air Cleaners to Improve Indoor Air Quality and COPD Health (CLEAN AIR, clinicaltrials.gov #NCT02236858, registration date 9/11/2014) and CURE COPD (Obesity and Adverse Dietary Patterns as Susceptibility Factors to Pollutant Exposure in Urban COPD), provided an opportunity to examine longitudinal anemia status in a well-phenotyped population of former smokers with moderate to severe COPD. Understanding the association between longitudinal anemia status trends and clinical COPD outcomes may help identify individuals with COPD who might benefit from anemia treatment.

## Methods

Former smokers with moderate to severe COPD [14] were recruited into two studies with similar inclusion criteria (age ≥ 40y, > 10 pack-years smoked, post-bronchodilator forced expiratory volume in one second [FEV_1_] to forced vital capacity [FVC] ratio less than 0.7 and FEV_1_ < 80% predicted). Briefly, the CLEAN AIR study was a double-blind randomized control trial of air cleaners with high efficiency particulate air and carbon filters with 5 study visits (pre-randomization, baseline, 3-month, 6-month, 9-month) over a 9-month period described in detail elsewhere [15]. Both pre- and post-randomization visits were included, and while the air cleaner intervention is thought to have minimal effect on anemia status, treatment arm was included as a covariate (see statistical analysis below). CURE COPD was an observational cohort study evaluating the interactive effects of indoor air pollution and diet, with 3 study visits (baseline, 3-month, 6-month) over a 6-month period. For 44 participants who were enrolled in both studies the CLEAN AIR data was included in the analysis if hemoglobin data was available from CLEAN AIR, otherwise available hemoglobin data from the CURE study was incorporated. Informed consent was obtained from all participants and studies were approved by the Johns Hopkins Institutional Review Board.

Complete blood counts and clinical outcomes were obtained at each visit. Anemia was defined as hemoglobin < 12 g/dL for women and < 13 g/dL for men [16]. Spirometry was performed according to the American Thoracic Society criteria [17] (Koko spirometer; nSpire). Questionnaires included the COPD Assessment Test [18] (CAT), modified Medical Research Council Questionnaire [19] (MMRC), St. Georges Respiratory Questionnaire [20] (SGRQ), and Ease of Cough and Sputum Clearance [21] (ECSC) score. Six-minute walk distance (6MWD) [22] was obtained in CLEAN AIR.

COPD exacerbations, assessed over the preceding 3-months of each study visit, were defined as (a) *moderate* if leading to use of antibiotics or steroids or unscheduled physician visit, or (b) *severe* if leading to an ED visit or hospitalization. Baseline CAD (history of heart attack and/or stent procedure) and chronic kidney disease (CKD) were assessed utilizing self-report. Non-contrast computed tomography (CT) scans of the chest were used to quantify CAC burden by Agatston score, quantified by a single fellowship-trained cardiothoracic radiologist (C.T.L.) as described previously [23]. 

Participant characteristics were compared between ever-anemic (at least one hemoglobin level below anemia threshold for the entire duration of the study) and never-anemic (all hemoglobin levels above the anemia threshold for the entire duration of the study) participants using *t-*tests and chi-squared tests. In the first analytic model, the association between time-varying anemia status and outcome was assessed by generalized estimating equations regression, accounting for repeated measures of observations within participants. With this approach, anemia status (as defined above) was determined separately at each time point. Gaussian distribution and identity link were used for the continuous outcomes while Poisson distribution and log link were used for the exacerbation outcomes with exchangeable working correlation structure and robust standard errors. The model was adjusted by baseline covariates including age, gender, race, education, tobacco pack-years, body-mass index, inhaler use, CVD (composite variable described previously [23]), supplemental oxygen use (continuous use with rest, exertion, and sleep), and study (including randomization status for CLEAN AIR). FEV_1_% predicted using the Global Lung Function Initiative race-neutral spirometry equation was used to adjust for baseline lung function, except when FEV_1_% predicted or absolute lung function (FEV_1_ best) was examined as an outcome. Effect modification by CAD burden on the association between anemia status and COPD outcomes was explored using CAC burden measured by the Agatston Score.

For participants with two or more hemoglobin measurements, using each participant’s serial anemia status observations (dichotomized), likelihood of developing anemia during the study period was profiled and assessed for association with changes in COPD morbidity. Participants with less than two anemia status observations during the study were excluded from this analysis. For exploratory analysis of the association between longitudinal anemia risk profiles and changes in COPD morbidity, a two-step approach was used. First, the change in each participant’s likelihood of anemia was estimated by unconditional linear regression of dichotomous anemia status on time (represented by study visit modeled continuously) using linear mixed model (LMM) with random intercept and slope [24]. As the random slopes in LMM regression represents the distinct growth rate of anemia probability for each participant, the estimated random slopes were exported into a separate post-hoc analysis [25, 26] in which the participants were divided into two groups of high versus low anemia risk-growth rate using the quartile cutoff (random slope ≥ 75th percentile or < 75th percentile). As there is no standardized threshold, sensitivity analysis was conducted using continuous growth rate, as well as the more stringent inter-quintile level (80th percentile) and inter-decile level (90th percentile) levels as cutoffs. Second, we assessed the association of the anemia risk profile with the changes in COPD outcomes by regressing the COPD outcome on the interaction between time and anemia risk profile using LMM regression with random intercept. The model was adjusted by the same covariates as above and by the outcomes’ baseline values. A three-way interaction term between time, anemia risk profile, and continuous Agatston score was used to explore effect modification by baseline CAC on the difference in COPD outcome trends by anemia risk growth rate profile.

## Results

### Patient characteristics

A total of 159 patients with at least one hemoglobin measurement were included in the study with a mean ± SD age of 66.5 ± 8.3 years and post-bronchodilator FEV_1_ (% predicted) of 51.4 ± 17.0%. Hemoglobin values ranged from 7.9 to 18.2 g/dL, with a mean ± SD of 12.9 ± 1.4 g/dL for women and 13.8 ± 1.7 g/dL for men. 41% of patients (*n* = 65) were anemic at one or more study visits – “ever-anemic”. Baseline characteristics were categorized by ever-anemia status (Table 1). Ever-anemia was associated with lower proportion of white participants (46% versus 64% in never-anemic participants, *p* = 0.03), lower baseline FEV_1_% predicted (48.2% versus 53.8%, *p* = 0.04), higher rate of CKD (10.8% versus 2.1%, *p* = 0.02), and higher mean number of cardiovascular comorbidities (0.7 ± 0.9 versus 0.4 ± 0.7, *p* = 0.01). Baseline COPD health status and exercise capacity did not differ between the two groups. Of the 151 participants who had two or more hemoglobin measurements, 26% (*n* = 40) had changes in anemia status during the study period while the remainder were consistently anemic (16%) or non-anemic (58%) throughout the entire study period (Table S1).

**Table 1:** Baseline participant characteristics by ever-anemia status during study period

	Never Anemic (*N* = 94)	Ever-Anemic (*N* = 65)	*P*-value
**Demographics**			
Age	66.9 (8.4)	66.0 (8.1)	0.49
Gender, N (% Female)	51 (54.3%)	31 (47.7%)	0.42
Race, N (% White)	60 (63.8%)	30 (46.2%)	0.03
Education, N (% Some college or above)	57 (60.6%)	31 (47.7%)	0.11
Pack-Years	50.0 (32.4)	48.7 (31.1)	0.80
BMI	31.3 (8.2)	32.0 (8.1)	0.61
ICS or LABA or LAMA, N (% Yes)	72 (76.6%)	49 (75.4%)	0.86
**Comorbidities**			
Cardiovascular Disease, (#)	0.4 (0.7)	0.7 (0.9)	0.01
Chronic Kidney Disease, N (%)	2 (2.1%)	7 (10.8%)	0.02
Agatston score^†^	612.5 (1237.7)	923.8 (1521.4)	0.20
Agatston Categorical^†^, N (%)			0.04
0	23 (27.7%)	6 (11.8%)	
0–99	24 (28.9%)	11 (21.6%)	
100–399	11 (13.3%)	8 (15.7%)	
≥ 400	25 (30.1%)	26 (51.0%)	
Hemoglobin	14.2 (1.1)	12.0 (1.1)	< 0.01
**Lung Function**,** Symptom Burden & Exacerbation History**			
FEV_1_% Predicted	53.8 (16.6)	48.2 (17.0)	0.04
COPD Assessment Test	17.7 (7,5)	19.0 (7.4)	0.32
St. George’s Respiratory Questionnaire	46 (18)	48 (15)	0.50
6-Minute Walk Distance (m)	256 (126)	206 (116)	0.08
Moderate/Severe Exacerbations in the Past 12 months, N (% Yes)	52 (55.9%)	36 (55.4%)	0.95
Supplemental oxygen use, N (% Yes)	18 (19.4%)	20 (30.8%)	0.10

Abbreviations: BMI = body-mass index, FEV_1_ = forced expiratory volume in one second, ICS = inhaled corticosteroid, LABA = long-acting beta agonist, LAMA = long-acting muscarinic antagonist

^†^*n* = 25 participants were missing observations for agatston

### Repeated measures of anemia status and clinical COPD outcomes

The association between repeated measures of anemia status and clinical COPD outcomes are shown in Table 2. After adjusting for baseline covariates, anemia (vs. non-anemia) was associated with 2.5 points higher SGRQ score (95% CI: 0.1,4.8, *p* = 0.04), lower 6MWD by 38.6 meters (m) (95% CI: -67.7, -7.44, *p* = 0.02), and 1.8 times higher moderate-to-severe exacerbations (95% CI: 1.1,2.8) during the study. Anemia was also associated with lower lung function by FEV_1_ percent predicted (-1.9% [95% CI: -3.3, -0.5], *p* = 0.01) and absolute FEV_1_ (-0.05 L [95%CI: -0.08, -0.01], 0.01). No statistically significant difference was seen for the other COPD outcomes (Table 2).

**Table 2:** Effect estimate of COPD outcomes between repeated measures of anemia versus non-anemia

	Mean Difference^†^ (95% CI)	*P*-value
CAT	0.08 (-1.3, 1.5)	0.91
MMRC	0.1 (-0.1, 0.3)	0.25
SGRQ	**2.5 (0.1**,** 4.8)**	**0.04**
CCQ	0.1 (-0.1, 0.3)	0.30
ECSC	0.3 (-0.5, 1.1)	0.47
BCSC	-0.1 (-0.4, 0.3)	0.64
6MWD, meters	**-38.6 (-67.7**,** -7.44)**	**0.02**
FEV_1_% Predicted*	**-1.9 (-3.3**,** -0.5)**	**0.01**
FEV_1_ Best**, L	**-0.05 (-0.08**,** -0.01)**	**0.01**
	**Incidence Rate Ratio** ^**‡**^ **(95% CI)**	**P-value**
Severe Exacerbations	1.9 (0.96, 3.7)	0.065
Moderate or Severe Exacerbations	**1.8 (1.1**,** 2.8)**	**0.02**

All models were adjusted by baseline covariates, including age, gender, race, educational attainment, smoking pack-years, BMI, medication use, supplemental oxygen use, CVD history, FEV_1_% predicted, and study

*For FEV_1_% predicted as an outcome, baseline FEV_1_% predicted was excluded as a covariate

**For FEV_1_ best as an outcome, height was included and FEV_1_% predicted was excluded as covariates

†The effect estimate represents the predicted mean difference in the continuous outcome level (e.g., CAT score) between anemic vs. non-anemic

‡Predicted incidence rate ratio (IRR) of the 3-month retrospective exacerbation (e.g., number of episodes of severe exacerbation in the 3-months prior to the clinic visit) between anemic vs. non-anemic

**Fig. 1:**
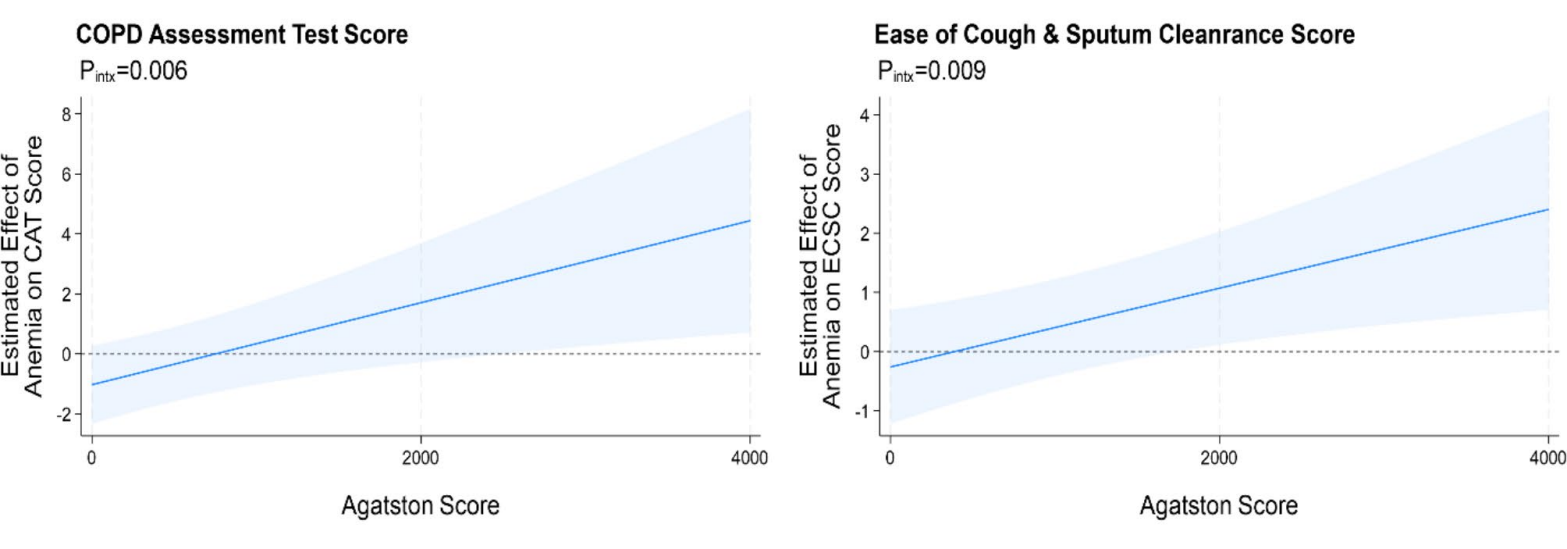
Predicted mean difference in symptom score with 95% confidence interval for repeated measures anemia vs. non-anemia, by CAC burden as measured by continuous baseline Agatston score. (Abbreviations: P_intx_=p-value for interaction term)

Similar results were observed with sensitivity analysis excluding individuals with polycythemia (defined as hemoglobin > 16 g/dL in females, > 16.5 g/dL in males; *n* = 9 ever-polycythemia). CAC burden by Agatston score was available for 134 patients (84%). Scores ranged from 0 to 8542 with median of 123 (Q1, Q3: 1, 810). There was evidence of effect modification by CAC burden on the association between anemia and COPD outcome such that at higher Agatston scores the mean difference in COPD outcome by anemia status was greater for both CAT score (P_interaction_=0.006) and ECSC score (P_interaction_=0.009) (Fig. 1).

The association between anemia with other COPD outcomes did not differ statistically significantly by Agatston score (Table S2).

### Association of longitudinal anemia risk profile and COPD outcomes over time

The 151 participants with anemia status observations at two or more visits during the study period were included in the longitudinal anemia risk profile analysis. The trajectory analysis cohort had a similar proportion of ever-anemic (42%) as did the primary analytic cohort (41%) and no significant difference in patient characteristics were observed between excluded and included participants other than prevalence of CKD (Table S3). Overall, anemia status was stable over the study period with the estimated median change in probability of anemia per year (random slope for time) of -0.0002 (Q1,Q3: -0.0001,0.0038). Thirty-seven out of 151 participants had high anemia-risk growth rates with random slope estimates at or above the 75th percentile level, corresponding to an increasing risk of anemia. The distribution of random slope estimates in those 37 individuals had a median (Q1,Q3) random slope of 0.025 (0.0002, 0.046) (Figure S1). In comparison, the remaining 114 participants with low anemia-risk growth rates, reflecting decreasing or stable risk of anemia over time, had a median (Q1,Q3) random slope of -0.0001 (-0.0028, 0.0022) (Figure S1). Participants with high anemia risk-growth rate (increasing anemia risk) were primarily those who developed anemia during the study period. Low anemia risk-growth rate participants (decreasing/stable anemia risk) consisted mostly of those with non-changing anemia status across all visits, either anemic or non-anemic.

**Fig. 2:**
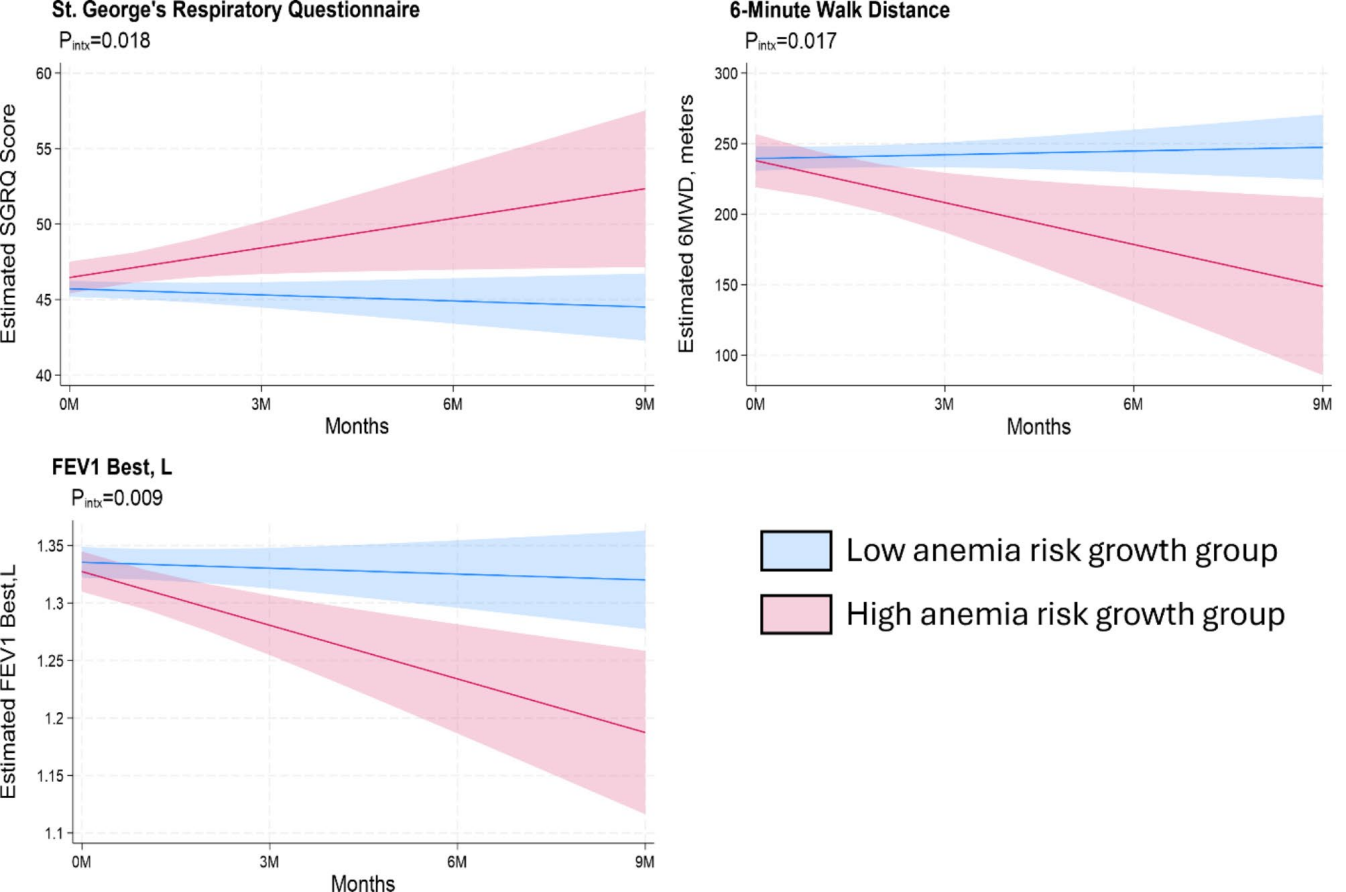
Comparison of estimated change in COPD outcomes over time between participants with high anemia risk-growth rate (*n* = 37) versus low anemia risk-growth rate (*n* = 114)

Comparing the two primary anemia risk-growth rate profiles, there was a significantly higher estimated rate of decline per 12-months in the FEV_1_ for participants with increasing anemia risk, with an average change over 12-months of -0.186 L (95%CI: -0.285,-0.088) versus − 0.020 L (95%CI:-0.078,0.037) for the decreasing/stable anemia risk participants (P_intx_ =0.009) and the 6MWD with an average 6-month change of − 119.0 m (95%CI:-213.2,-24.7) versus 10.7 m (95%CI: -24.1,45.4) (P_intx_ =0.017) (Fig. 2**)**.

A similar trend was observed for the SGRQ score increasing on average over 6 months by 7.8 points (95%CI: 0.51,15.2) for increasing anemia risk participants compared to largely unchanged at -1.6 points (95%CI: -4.6,1.4) for participants with decreasing/stable anemia risk (P_intx_ =0.018) (Table S4). There was no effect modification by CAC on differences in COPD outcome trends by anemia risk growth rate profile as measured by a three-way interaction between time, anemia risk growth rate profile, and continuous Agatston score (data not shown).

Sensitivity analyses using continuous measure of growth rate, as well as inter-quintile (80th percentile) and inter-decile (90th percentile) cutoff levels showed largely similar results as with the 75th percentile cutoff, with statistically significant interactions between time and anemia risk profile for FEV_1_, 6MWD, and SGRQ trajectories, except for SGRQ when using inter-decile cutoff and for 6MWD when using continuous measure (data not shown).

## Discussion

In this study serial repeated measures of anemia status are demonstrated to be associated with increased COPD morbidity, including worse quality of life, lower functional status by 6MWD, and higher rates of severe and moderate-to-severe exacerbations. Furthermore, participants with increasing risk of developing anemia over time demonstrated a more rapid decline in functional status, lung function, and symptom burden compared to those with stable or decreasing risk of anemia. Together these findings suggest that longitudinal anemia status may be a predictor of morbidity and disease trajectory in COPD, with potential to identify the COPD sub-population which may benefit from treatment of anemia, supporting the need for guidance in work up and management of anemia in COPD.

We present novel findings examining the relationship between anemia status and COPD outcomes by repeated-measures analysis, adjusting for key confounders including cardiovascular comorbidity and supplemental oxygen use, which corroborate prior studies examining a single measurement of hemoglobin and noted associations with COPD morbidity [4–8]. Our findings demonstrated that SGRQ and 6MWD, two outcomes that take into account functional limitations and quality of life that plausibly can be impacted by other comorbidities, were associated with anemia. Alternatively, this association may be explained by the fact that anemia, unless in a very severe range, could manifest as exercise intolerance and decreased quality of life without a direct impact of dyspnea or other respiratory symptoms. Specifically, oxygen carrying capacity is reduced with anemia which limits oxidative capacity in muscle, leading to reduced exercise capacity, physical activity and mobility. In addition, a recently published study on cause-specific mortality in COPDGene, a longitudinal cohort of tobacco cigarette users including those with COPD, highlighted the importance of respiratory quality of life not only as a determinant of all-cause but also respiratory-specific mortality [27]. 

Anemia was also associated with increased exacerbations, another important predictor of mortality in COPD. A few previous studies have demonstrated anemia to be associated with higher rates of severe exacerbations and COPD exacerbations requiring hospitalization [28, 29]. As a corollary, our group found that polycythemia is associated with a reduced rate of severe exacerbations despite polycythemic individuals having worse disease severity by FEV_1_% predicted and increased emphysema [6, 30]. This trend may be explained by hemoglobin’s role as the limiting factor for oxygen carrying capacity [31]. Although the models in this study adjusted for lung function and supplemental oxygen use, anemia may result in reduced reserve to handle COPD exacerbations due to lower capacity for oxygen delivery which may compound symptoms into a more overt exacerbation. Although reverse causality for the association between chronic conditions like anemia and COPD exacerbations cannot be excluded, given the consistent epidemiological evidence, anemia may be an important treatable risk factor for COPD exacerbations and hospitalization.

This study further explored longitudinal anemia status trends using anemia risk profiles which has not yet been studied in the COPD population. Participants with increasing anemia risk had evidence of worse COPD disease trajectory by multiple parameters even compared to individuals with a stable anemia risk profile, which included persistently anemic individuals. Thus, it may not be anemia per se but the development of anemia, represented by increasing anemia risk, that is more relevant to COPD disease trajectory. The profile of increasing anemia risk could represent subacute blood loss such as a gastrointestinal bleed leading to iron deficiency or a state of reduction in bone marrow function as can be seen in states of malnutrition. It may also represent a significant worsening of existing or new multimorbidity, for example a new malignancy, reflecting an overall adverse trajectory of health. In both situations there may be a greater effect on COPD disease trajectory compared to a state of stable anemia risk, often seen with anemia of chronic disease, where an individual may be chronically compensated for the anemic state and thus more protected against adverse outcomes.

This pattern is reflected in the varying approaches to treatment of anemia in other chronic diseases. In the heart failure population where anemia was similarly found to be associated with poor outcomes, recent studies have underscored of the importance of not only iron deficiency anemia but also iron deficiency irrespective of anemia status [32]. Clinical trials have shown improvement in symptom severity, exercise tolerance and reduction of hospitalizations among heart failure patients treated for iron deficiency with intravenous iron [33]. Targeting iron deficient patients even prior to development of anemia may be selecting for patients who have the profile of increasing anemia risk which is more likely to affect disease status and symptom burden.

Finally, anemia and COPD do not co-exist in a vacuum but must be considered in the context of real-life COPD with multimorbidity and a high prevalence of cardiovascular comorbidities [1]. Yet the assessment of comorbid diseases in clinical studies remains challenging due to the reliance on participant self-report which, even when combined with medical record and medication review, are often discordant with diagnoses determined by objective disease criteria [34]. To overcome this, the Agatston score calculated from chest CTs provided an objective, graded measure of CAC burden which allowed for a more robust analysis to understand the presence of effect modification. Studies in the general population have shown an Agatston score of 300 to 400 is associated with an approximately five-fold increase in mortality compared to a score of 0 [35]. In COPD, CAC is more prevalent and the degree of CAC burden is greater, when compared with smokers with normal spirometry or non-smokers [36]. In this study, the statistically significant interactions between CAC and anemia were observed in the ranges of very high Agatston scores (≥ 2000) which may appear less clinically relevant. However, the Agatston score cut-off most predictive of the presence of CAD, myocardial infarction and heart failure (determined retrospectively) in a COPD population, has been suggested to be 1500 as opposed to 400, the accepted threshold defining severe CAC in the general population [37]. Of note, effect modification by CAC was limited to CAT scores and ECSC, which may be reflective of greater correlation of anemia, CAD, and respiratory symptoms in the chronic bronchitis COPD phenotype which is considered the more inflammatory sub-phenotype [38]. Overall, this analysis, while still exploratory with limited sample size, suggests that individuals with COPD with significant CAD based on high CAC burden may be a specific phenotype that can obtain greater benefit from treatment of anemia.

The associations of anemia, CAD, and COPD morbidity may be a manifestation of multimorbidity or driven by a unique causal mechanism. Prior work in the SPIROMICS observational cohort of individuals with COPD demonstrated that the effect of anemia on poor outcomes was independent of the impact of overall comorbidity burden, but the association between anemia and poor outcomes was stronger among participants with a higher burden of cardiometabolic comorbidities [5]. Further evidence that anemia is not just a marker of multimorbidity was shown in the study by Vanfleteren and colleagues which characterized clustering patterns of comorbidities in COPD including anemia. The comorbidity clusters revealed the prevalence of atherosclerosis was highest among individuals with anemia, but anemia tended to cluster in the “less-comorbid” group and did not correlate with the total burden of comorbidities [39]. Ultimately, while it is unclear whether this phenomenon reflects multimorbidity generally, the results underscore the potential value of screening for and treating anemia in COPD, given the evidence-based benefit of treating anemia and iron deficiency in conditions such as congestive heart failure which is similarly accompanied by a high comorbidity burden.

This present study has several limitations. First, this is a single center study comprised of two studies of former smokers with a relatively small sample size. This may limit generalizability of the results to the population with COPD actively smoking, have never smoked, or have different causative etiologies for COPD. Second, the study period was 6 to 9 months, a relatively short period for evaluation of trajectories, which does not allow for assessment of the importance of longer-term changes in anemia progression.

In conclusion, despite growing evidence of the importance of comorbidities in COPD outcomes and in understanding different COPD phenotypes, we lack evidence that could inform specific guidance for the approach to screening for and treatment of comorbidities in COPD. Our study adds to the growing evidence of the importance of anemia, specifically longitudinal anemia status, on COPD morbidity and disease trajectory. A greater understanding of the types of anemia as well as causal relationship between anemia and COPD outcomes will be necessary to elucidate the best approach to treatment. In the future, investigating the etiology of anemia in the COPD population in combination with longitudinal anemia status will help to identify patients who are both at risk for greater morbidity from anemia and thus may benefit the most from screening and treatment.

## Electronic Supplementary Material

Below is the link to the electronic supplementary material.

Supplementary Material 1

## Data Availability

The datasets used and/or analyzed during the current study are available from the corresponding author on reasonable request.

## Abbreviations

CAC: Coronary artery calcium
CAD: Coronary artery disease
CAT: COPD Assessment Test
CKD: Chronic kidney disease
CT: Computed tomography
CVD: Cardiovascular disease
ECSC: Ease of Cough and Sputum Clearance
FEV_1_: Forced expiratory volume in one second
FVC: Forced vital capacity
LMM: Linear mixed model
MMRC: Modified Medical Research Council Questionnaire
P_intx_: P-value for interaction term
SGRQ: St. Georges Respiratory Questionnaire
6MWD: Six-minute walk distance

## Declarations

### Clinical trial number

Not applicable. However, part of the data utilized in this study was obtained from a previously completed clinical trial: CLEAN AIR, clinicaltrials.gov #NCT02236858, registration date 9/11/2014.

## Study abbreviations

CLEAN AIR: Clinical Trial of Air Cleaners to Improve Indoor Air Quality and COPD Health
COPDGene: Genetic epidemiology of COPD study
CURE COPD: Obesity and Adverse Dietary Patterns as Susceptibility Factors to Pollutant Exposure in Urban COPD
SPIROMICS: Subpopulations and Intermediate Outcome Measures in COPD Study
