# Supplementary material for: Longitudinal anemia status and risk for adverse outcomes in former smokers with COPD
Source: Respir Res. 2024 Dec 19;25:437. doi: 10.1186/s12931-024-03071-y (PMC11660789; doi:10.1186/s12931-024-03071-y)
Supplement: Supplementary file 1 — Supplementary Material 1 [file 12931_2024_3071_MOESM1_ESM.docx]

**Supplemental Table S1:** Distribution of anemia status patterns throughout study period for participants with two or more hemoglobin measurements.

| **Anemia Status Pattern** | **n (%)** |
| --- | --- |
| **Stable during study period** | |
| Stable Non-Anemic | 87 (58%) |
| Stable Anemic | 24 (16%) |
| **Transition during study period** | |
| Non-Anemic to Anemic | 11 (7.3%) |
| Anemic to Non-Anemic | 11 (7.3%) |
| Fluctuating status | 18 (11.4%) |

**Supplemental Table S2:** Effect Estimate of Repeated Measures of Anemia (vs. Non-Anemia) on COPD Outcomes by Baseline CAC Burden as Measured by Continuous Agatston Score

|  | Mean Difference†  (95% CI) | | 2-way  Interaction^£^ |
| --- | --- | --- | --- |
|  | Agatston=0 | Agatston=2000 | P-value |
| CAT | -1.03 (-2.3, 0.3) | **1.7 (-0.3, 3.7)** | **0.006** |
| MMRC | 0.11 (-0.1, 0.3) | 0.02 (-0.2, 0.3) | 0.492 |
| SGRQ | 1.6 (-1.3, 4.4) | 4.1 (0.5, 7.6) | 0.196 |
| CCQ | 0.08 (-0.2, 0.3) | 0.0 (-0.2, 0.2) | 0.441 |
| ECSC | -0.26 (-1.2, 0.7) | **1.1 (0.1, 2.0)** | **0.009** |
| BCSC | -0.07 (-0.5, 0.3) | 0.1(-0.4, 0.6) | 0.631 |
| 6MWD, meters | -57.0 (-91.3, -22.8) | -53.6 (-90.3, -16.9) | 0.820 |
| FEV1 % Predicted* | -1.8 (-3.6, -0.07) | -1.8 (-4.2, 0.6) | 0.856 |
|  | Incidence Rate Ratio‡  (95% CI) | | 2-way  Interaction^£^ |
|  | Agatston=0 | Agatston=2000 | P-value |
| Severe Exacerbations | 2.41 (1.1, 5.2) | 1.9 (0.9, 4.0) | 0.537 |
| Moderate or Severe Exacerbations | 1.9 (1.1, 3.2) | 1.4 (0.7, 2.6) | 0.324 |

*All models were adjusted by baseline covariates, including age, gender, race, educational attainment, smoking pack-years, BMI, medication use, CAD risk factor, supplemental oxygen use, FEV1 % predicted, and study.*

**For FEV1 % predicted as an outcome, the covariate list excluded the baseline FEV1 % predicted.*

*†The effect estimate represents the predicted mean difference in the continuous outcome level (e.g., CAT score) between anemic vs. non-anemic while holding constant the baseline agatston at a fixed level in the interaction regression analysis (e.g., agatston=0).*

*‡The effect estimate represents the predicted incidence rate ratio (IRR) of the 3-month retrospective exacerbation (e.g., number of episodes of severe exacerbation in the 3-months prior to the clinic visit) between anemic vs. non-anemic while holding constant the baseline agatston at a fixed level in the interaction regression analysis (e.g., agatston=0).*

*£ The p-value represents the statistical significance of the effect estimate of the 2-way interaction between anemia and baseline agatston on COPD outcome.*

**Supplemental Table S3:** Comparison of baseline characteristics of participants excluded versus included in anemia risk profile analysis

|  | All  (N=159) | Excluded from Trajectory Analysis  (N=8) | Included in Trajectory Analysis  (N=151) | P-value |
| --- | --- | --- | --- | --- |
| Age | 66.5 (8.3) | 68.5 (5.9) | 66.4 (8.4) | 0.493 |
| Gender, N (% Female) | 82 (52%) | 5 (62%) | 77 (51%) | 0.526 |
| Race, N (% White) | 90 (57%) | 3 (38%) | 87 (58%) | 0.263 |
| Education,  N (% Some college or above) | 88 (55%) | 4 (50%) | 84 (56%) | 0.755 |
| Pack-Years | 50 (32) | 42 (23) | 50 (32) | 0.508 |
| BMI | 32 (8.2) | 30 (8.0) | 32 (8.2) | 0.572 |
| ICS or LABA or LAMA, N (% Yes) | 121 (76%) | 4 (50%) | 117 (76%) | 0.076 |
| Hemoglobin | 13.3 (1.6) | 14.2 (1.2) | 13.3 (1.6) | 0.133 |
| Ever-anemia | 65 (41%) | 1 (13%) | 64 (42%) | 0.094 |
| Cardiovascular Disease, (#, SD)† | 0.5 (0.8) | 0.8 (1.0) | 0.5 (0.8) | 0.392 |
| Kidney Disease, N (% Yes) | 9 (5.7%) | 2 (25%) | 7 (4.6%) | 0.015 |
| FEV1 % Predicted | 51.4 (17) | 57.4 (17) | 51.1 (17) | 0.308 |

Abbreviations: BMI=body-mass index, FEV_1_=forced expiratory volume in one second, ICS=inhaled corticosteroid, LABA=long-acting beta agonist, LAMA=long-acting muscarinic antagonist

^†^number indicates number of cardiovascular comorbidities and standard deviation

**Supplemental Table S4:** Estimated change in COPD outcome (per 12 months) by estimated anemia risk growth profile

|  | Mean Difference*†*  (95% CI) | | 2-Way Interaction |
| --- | --- | --- | --- |
|  | Low Anemia Risk  Growth Rate | High Anemia Risk  Growth Rate | P-value*^‡^* |
| CAT | -3.0 (-4.4, -1.5) | 0.58 (-3.1, 4.2) | 0.070 |
| MMRC | 0.13 (-0.1, 0.4) | 0.15 (-0.4, 0.6) | 0.944 |
| SGRQ | -1.6 (-4.6, 1.4) | 7.8 (0.5, 15.2) | **0.018** |
| CCQ | -0.26 (-0.4, -0.1) | -0.19 (-0.6, 0.3) | 0.768 |
| ECSC | -1.1 (-1.9, -0.2) | -0.5 (-2.3, 1.3) | 0.604 |
| BCSC | -0.73 (-1.2, -0.3) | -0.3 (-1.1, 0.6) | 0.391 |
| 6MWD, meters | 10.7 (-24.1, 45.4) | -119.0 (-213, -24.7) | **0.017** |
| FEV1 % Predicted* | -0.5 (-2.8, 1.9) | -6.0 (-10.6, -1.4) | **0.056** |
| FEV1 best (L)** | -0.020 (-0.078, 0.037) | -0.186 (-0.425, -0.088) | **0.009** |
|  | Incidence Rate Ratio  (95% CI) | | 2-Way Interaction |
|  | Anemia Risk  Low Growth Rate  Group | Anemia Risk  High Growth Rate  Group | P-value |
| Severe Exacerbations^£^ | -0.006 (-0.3, 0.3) | -0.25 (-1.3, 0.85) | 0.544 |
| Moderate or Severe^£^  Exacerbations | -0.059 (-0.3, 0.2) | 1.6 (0.1, 3.1) | **0.003** |

*All models were adjusted by baseline covariates, including age, gender, race, educational attainment, smoking pack-years, BMI, medication use, supplemental oxygen use, CVD history, FEV1 % predicted, and study, as well as two-way interaction with each covariate.*

**For FEV1 % predicted as an outcome, the covariate list excluded the baseline FEV1 % predicted and its two-way interaction with time.*

***For FEV1best height was included as a covariate in place of baseline FEV1 % Pred*

*†The effect estimate represents the predicted mean difference in the continuous outcome level (e.g., CAT score) between anemic vs. non-anemic while holding constant the anemia risk profile at a fixed level in the interaction regression analysis (e.g., anemia risk profile = high growth rate group).*

*£ The coefficient for exacerbation outcome represents the estimated annual change in mean 3-month exacerbation count -- i.e., the change in the mean # of exacerbations within 3-month period that is expected after one year.*

*^‡^The p-value represents the statistical significance of the effect estimate of the 2-way interaction between time and anemia risk profile on COPD outcome.*

**Supplemental Figure S1:** Predicted level of probability of anemia based on unconditional linear probability model using generalized linear mixed model regression of anemia status on time.

**LOW ANEMIA RISK HIGH ANEMIA RISK**

**GROWTH RATE GROWTH RATE**
